# Simulation in physiotherapy students for clinical decisions during interaction with people with low back pain: randomised controlled trial

**DOI:** 10.1186/s12909-021-02812-7

**Published:** 2021-07-09

**Authors:** Carolina Sandoval-Cuellar, Margareth Lorena Alfonso-Mora, Adriana Lucia Castellanos-Garrido, Angélica del Pilar Villarraga-Nieto, Ruth Liliana Goyeneche-Ortegón, Martha Lucia Acosta-Otalora, Rocío del Pilar Castellanos-Vega, Elisa Andrea Cobo-Mejía

**Affiliations:** 1 Universidad de Boyacá, Tunja, Colombia; 2 Universidad de La Sabana, Chía, Colombia; 3 Center of Clinical Simulation, Universidad de La Sabana, Chía, Colombia

**Keywords:** Simulation, Physiotherapy, Clinical skill, Simulated patient

## Abstract

**Background:**

Low back pain (LBP) is a condition with a high global prevalence, which is getting health professionals’ attention, including physiotherapists as they must have the skills to provide treatment that increases the patient’s quality of life. Clinical simulations could be a pedagogic strategy that facilitates adequate training for students to acquire skills to improve their professional reasoning in this clinical situation.

**Objective:**

This study sought to determine the effects of clinical simulations with simulated patients (SP) on the physiotherapy students’ clinical decision-making within a role-playing (RP) scenario while caring of LBP patients.

**Methods:**

This experimental study included 42 participants from two Colombian universities, randomized into two groups (SP, n = 21; RP, n = 21). The clinical skill of performing the Objective Structured Clinical Examination (OSCE-LBP) was evaluated while students cared for patients with LBP; after that, a pedagogical method was conducted that included clinical simulation and, finally, the OSCE-LBP was applied again to compare both groups.

**Results:**

Changes occurred in the OSCE-LBP among both groups of students: the scores increased (0.66 and 0.59 in RP and SP, respectively), and neither of the two was superior (p value 0.01; 95%CI − 0.21 to 0.23).

**Conclusion:**

Both types of simulation favor decision-making in professional reasoning in physiotherapy students during interactions with individuals with LBP.

*Trial registration*https://clinicaltrials.gov/ct2/show/NCT04428892 Identifying number: NCT04428892. It was retrospectively registered.

## Background

Low back pain (LBP) is one of the principal reasons for consulting health services globally [1, 2]. Epidemiological reports state that the incidence of this symptom is between 60 and 90% in the Western population, affecting almost 80% of people at least once in their lives [3]. LBP is considered one of the principal causes of absenteeism and the risks associated with it are diverse, including lifestyle, work and emotional factors, among others [3, 4]. In physiotherapy, it is the most frequent reason for care [1], which is why professionals must have the necessary skills to offer those people who consult them a comprehensive approach that minimizes reoccurrence and improves their quality of life. To achieve this, it is necessary for physiotherapy programs to provide sufficient tools to prepare future professionals treating people with LBP.

For this interaction, physiotherapists must: (1) perform physiotherapeutic examinations and evaluations, including interviews, systems reviews, and the selection and application of tests and measurements; (2) discuss the examination findings, supported by professional reasoning; (3) formulate physiotherapeutic diagnosis and prognosis in line with the findings; (4) provide information to patients about a treatment plan according to the examination findings; (5) execute the treatment plan supported by scientific evidence; and (6) demonstrate patient-centred treatment and communication skills during the physiotherapeutic interaction [5].

The aforementioned elements are closely related to decision-making clinical skills during the physiotherapeutic practice [6], such as professional reasoning, which is supported by knowledge and values originating from high levels of quality-focused patient care [7]. This is a process of applying integrated knowledge that includes intuition and permits timely development of examinations or physiotherapeutic interventions [7], through constant reflection during professional interactions.

Simulations can be used as a pedagogic tool that enables students to hone their treatment decision-making skills for patients with LBP. It is a potential strategy for developing diverse skills focused on the safety of the patient and the therapist [8]. Moreover, it allows the inclusion of simulated patients [9]; these are people who act out a specific health condition to facilitate teaching interpersonal and/or clinical skills [10]. Simulations provide students with an authentic learning experience [10] and, given their closeness to the real scenarios, facilitate effective student performance evaluation.

Clinical simulations allow students to get involved in direct interaction with what they are studying, rather than an intellectual observation or description, and enables them to make significant progress in their development through their own experience [11]. The World Confederation for Physical Therapy (WCPT) states that students must be guaranteed clinical interaction opportunities, which should always be accompanied by qualified professionals. Simulations may be linked to this process, given that controlled scenarios promote clinical reasoning, and theoretical information can be used to consolidate learning [12].

The evidence around simulations suggest that this is an educational strategy that increases teamwork capabilities, and it should be aligned with the principles of interprofessional education [13, 14]. Clinical simulations also promote core values, communication skills among professionals and patients, and patient safety [15–18]. Simulations in physiotherapy have been widely used for cardiorespiratory practice for training specific manual skills and decision-making processes in intensive care units. Students trained with simulation develop better results in manual and instrumental procedures in general [19–25].

Additionally, clinical simulations have demonstrated that students improve ‘confidence in clinical skills, clinical decision-making, treatment preparation and planning, communication skills, evaluating and modifying interventions, and interprofessional practice’ [26].

Therefore, clinical simulations were used to generate a controlled scenario with LBP simulated patients, seeking students to put into practice the acquired knowledge to make clinical decisions and respond to these patients’ needs within a particular context. Thus, this study aimed to determine the effects of clinical simulations with standardised patients on clinical skills decision-making of physiotherapy students while caring for LBP patients.

## Methods

### Design

A randomised controlled trial was performed with physiotherapy students from two Colombian universities. The students were randomised into two clinical simulation groups, simulated patients (SP) and role-playing simulations (RP). The study was approved by the ethics committee at the University of Boyacá under record number 164 on 09 June 2016, and by the subcommission on research and ethics at the University of La Sabana. The study was considered minimum risk and the students were assured that the research process would not affect their semester grades; hence, the evaluations were of a formative nature.

### Subjects

The study included 42 fifth-semester students from two physiotherapy programmes in two Colombian universities, based on the formula [27], in which $n=$ size per group, ${Z}_{1-\frac{\alpha }{2}}=$ normal two-tail standard deviation, $d=$ acceptable difference, and $s$ = standardised standard deviation of both comparison groups.

$$n = 2\frac{{\left[ {\left( {Z_{{1 - \frac{\alpha }{2}}} + Z_{\beta } } \right)^{2} } \right]}}{{d^{2} }}~s^{2}$$

At the time of the study, the participants had taken a total of 87 credits and were actively enrolled in the participating programs. The study excluded students < 18 age, those who had been internally or externally transferred from another academic program in health, exchange students, and those repeating and with prior experience in simulated practice in other assignments.

**Fig. 1:**
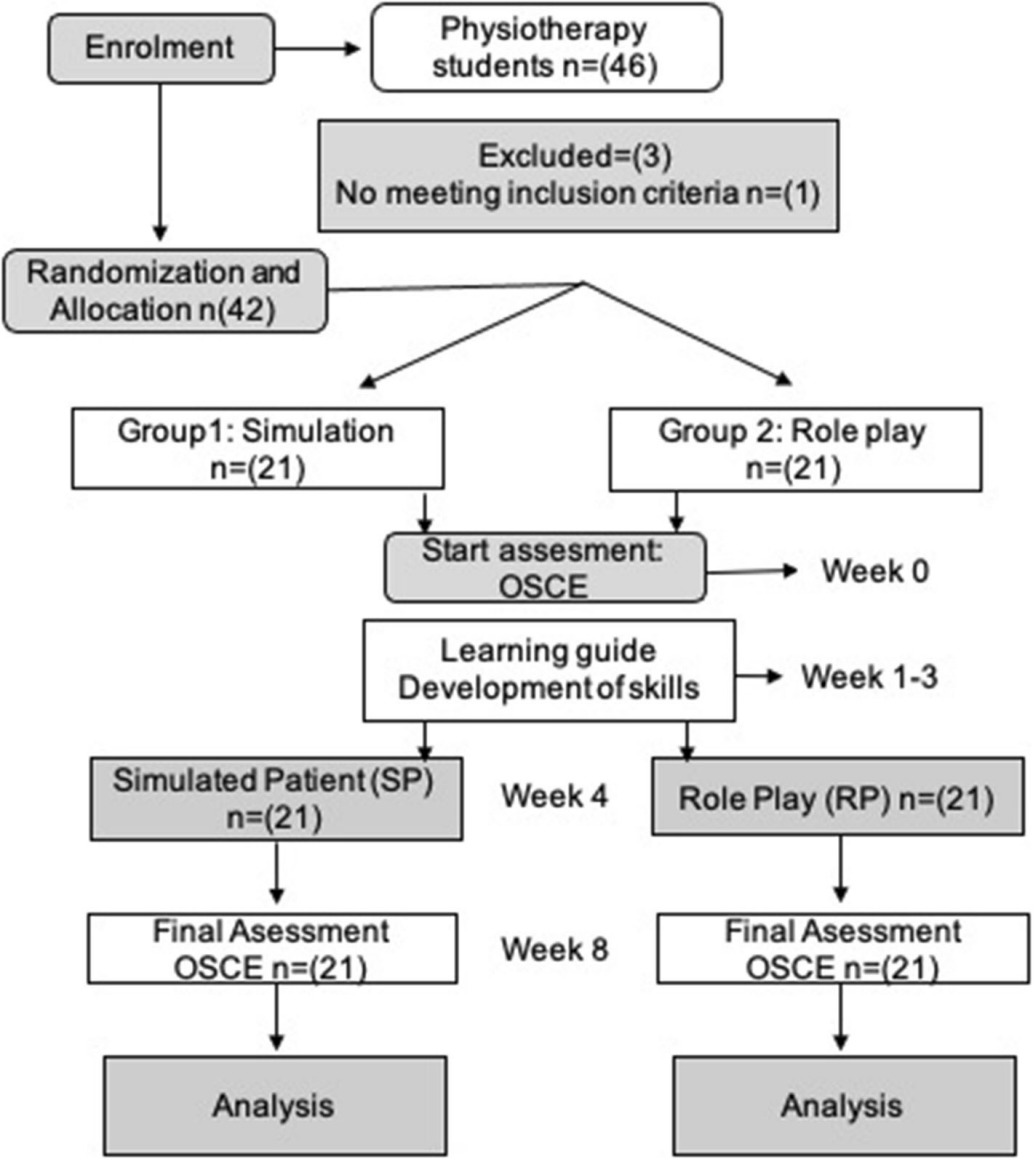
Flow diagram of the study ‘Simulated practice in physiotherapy students from the University of Boyacá and University of La Sabana on decision-making in clinical skills while caring for a person with LBP’. Intervention group: simulated practice (SP); control group: ‘role-playing’ (RP)

The study established stratified sampling by blocks in each university using the Epidat program version 3.1 and assigning participants to one of the SP and RP groups, a procedure overseen by a professor not involved with the study. A record of the process was kept (Fig. 1).

### Results measurements

The study considered the following variables: gender, percentage of attendance of academic activities in SP and RP, level of bilingualism, score on the Colombian ‘SaberPro’ state examination, and participants’ accumulated academic grade point average for their undergraduate formation.

The main outcome of this study was clinical reasoning assessed with the Objective Structured Clinical Examination for low back pain (OSCE-LBP). It was applied in a blinded format at the start (week 0) and end of the period for the SP and RP interventions (week 8) using the same case. This instrument was subjected to a content validity process with external experts in the study area [28]. Application of the OSCE-LBP, as an evaluation process, was conducted by professors that were external to the research process to minimise evaluation bias. OSCE-LBP training was carried out through systematic training in developing the OSCE-LBP in its different stages. Additionally, inter-rater correlation coefficient values were obtained [28], and the stages in which the professors had their best performance was identified compared with the denominated gold-standard evaluator [29].

The OSCE-LBP evaluates decision-making in clinical skills while caring for a person with LBP. It has seven stages, each with a specific weight, and assesses the skills or abilities described by the researchers regarding physiotherapeutic decision-making for a person with LBP, the stages are as follows:

1. Physiotherapeutic examination (stages 1, 2, and 3)
  I. Anamnesis: recognises the symptoms or antecedents, including red flags and personal aspects that can guide physiotherapists’ decision-making in relation to LBP.
  II. Systems Review: recognises physical symptoms that indicate red flags or signs of peripheral nerve involvement, or signs of LBP.
  III. Tests and measurements: applies tests and measurements related to mobility, articular integrity, strength of the lumbopelvic complex, and the integrity of the peripheral nerves of the lumbopelvic region.
2. Physiotherapeutic evaluation (stage 4)
  I. Explains to a person with LBP whether their condition is related to nociceptive LBP, neuropathic LBP, or another type (central sensitisation).
3. Diagnosis, prognosis, and intervention plan (stage 4)
  I. Proposes reachable objectives according to the characteristics of the person’s LBP.
  II. Provides information to the patient about the treatment plan to follow according to the findings obtained in the examination related to LBP prognosis.
  III. It is clear with the language used and in line with previous aspects of the interaction process.
4. Intervention (stages 5, 6, and 70,029)
  I. Includes therapeutic exercise according to the person’s needs and capacity.
  II. Includes manual techniques according to the needs.
  III. Includes specific physiotherapy techniques according to the needs.
  IV. Includes interpretation of scientific evidence that supports the decision-making process [30–36].
5. Demonstrates patient-centered care and communication skills during the process of professional interaction (all stages).
  I. Verbal and non-verbal communication includes touch and proximity, facial expression, eye contact, gesture and posture, observation, and listening use of silence [37].

The results method of scoring the OSCE-LBP was established on a scale from 0.0 to 5.0. This scoring was obtained with a specific weight for each stage. It was given by a research team in percentages as follows: stage 1, 17%; stage 2, 12%; Stage 3, 16%; stage 4, 15%; stage 5, 15%; stage 6, 13%; and stage 7%, 12%. An equivalency success was determined in the SP and RP interventions with a difference of 0.8 [38].

### Intervention

Initially, in the intervention of both groups, we developed a pedagogic process that was established to achieve the skills required to care for people with LBP in the following manner:

I. Learning guide process of therapeutic interaction with a person with LBP (LG-LBP). The guide was given to students participating 2 weeks before they were assigned to the SP or RP. The LG-LBP intention was to serve as a facilitator of the following phases of the learning process. The guide was constructed by the research team. It includes information and independent activities for students to conduct a search for physiotherapeutic examinations and treatment for people with LBP, supported by scientific evidence.
II. Development of skills through laboratory workshops for the prescription and application of manual techniques and therapeutic exercise for the health condition studied. Each session sought to develop the practical aspects of the learning guide (LG-LBP), which include the manual technique of therapeutic massage [30], and superficial technique of myofascial release for paravertebral muscles and those related to the lumbopelvic complex [39]. The exercise techniques included central stabilisation exercises [40], analytic stretching, and postural re-education principles [41]. We developed this activity in five sessions for 2 h each.

Subsequently, the students were randomised into two groups, SP and RP. The SP group consisted of clinical simulations with simulated patients, received in a class session, for clinical skills decision-making practice in caring for a person with LBP. Each session lasted approximately 120 min, with eight students per session, and the clinical case used for the SP sessions was subjected to face validity with experts in the area of study. Application of the simulated practice was conducted using the following sequence of elements:

- Context of the scenario: standardised for a clinical scenario of external consultation for the care of a person with LBP using physiotherapy.
- Simulation patient training: based on the clinical case elaborated and validated. A script was created for the actor to interpret a person with LBP. Three simulated patients were trained.
- Guiding questions: the professor asked guiding questions supported by the LG-LBP to have students reflect on the clinical situation observed. Students were asked to imagine other clinical situations and how the intervention would vary according to this.
- Concrete experience: students interacted with the simulated patients to carry out the reasoning process and thus provide care to them.
- Debriefing: at the end of the SP, a meeting was held based on reflective conversation about the learning experience from the clinical simulation with simulated patient in which students and professor discussed the positive aspects and the learning opportunities. This process was mediated by dialogue and active listening. Students reflected on what they had done, their feelings and emotions, what was opportune, and what they would improve for subsequent opportunities with a person with LBP. This type of data was not used in the analysis of this study.

The RP group, in turn, received a class session based on a ‘role-playing’ simulation strategy for LBP patients. This session lasted approximately 120 min, and the learning environment was the classroom in which students assumed different roles. Some acted like people with LBP and others as physiotherapists. The role-playing strategy did not include specific training for acting like a patient. The study cases used for the RP group were different from the SP group but had a similar level of complexity in LBP. The sequence of elements taken into account in the RP group was:

- Division of the students into two groups: one physiotherapist and one patient.
- Three different case studies were given to students. Case one was about pregnancy LBP, while case two included posttraumatic LBP, and case three was about nonspecific LBP.
- The students started the acting process as physiotherapists or patients.
- At the end of the session, the students guided by a professor closed the simulation with a conclusion for each case study, in a non-extensive way, like the debriefing process in the SP group.

### Statistical analysis

The purpose of the Shapiro–Wilks-Test was to demonstrate the normal distribution of the data. Then, descriptive results were presented as median and interquartile range (IQR) for ordinal Likert scale variables, and percentages for the nominal ones. The quantitative variables were the weighted average by academic credits, which value ranges from 0.0 to 5.0 according to the Colombian educational system (1 credit is equivalent to 48 h a week in academic activities), bilingualism, and age.

An intention-to-treat analysis was conducted to compare both groups, SP and RP. The OCSE-LBP scores were compared between baseline and post-intervention inside each group (SP and RP). In addition, the student's t-test for related samples was used to estimate differences. Finally, a hierarchical generalized linear model, with two levels of random intercepts and fixed coefficients (level 2: university; level 1: student) was implemented to estimate the effect of PR compared to SP on OCSE-LBP at the end of the intervention. In the linear model, ${y}_{ij}$ corresponds to the measurement of the OCSE-LBP after the pedagogical intervention in person ij; ${Y}_{1ij}$ corresponds to the baseline measurement in person ij; ${T}_{ij}$ is the treatment indicator variable (SP or RP); ${\beta }_{0}$ is the effect of the RP; ${\beta }_{1}$ is the effect of the baseline measurement; ${\beta }_{2}$ is the effect of the intervention and the focus of interest of the analysis; ${U}_{oj}$ is the differential of each university.

$$y_{{ij}} = \beta _{0} + \beta _{1} Y_{{1ij}} + \beta _{2} T_{{ij}} + U_{{oj}} + e_{{ij}}$$

To adjust for the baseline of the OCSE-LBP was applied because the comparison of the means between the study groups taking into account the baseline values generates a statistical power gain [42]. A confidence level of 95% was used in the estimation of parameters. Analyzes were performed using STATA version 14.

## Results

The participating population was mostly female, with a mean age of 21 years, an academic average of 3.6/5, and an average of 87 credits taken in the Physiotherapy programme at each university (Table 1).

**Table 1:** Characteristics of the participants

	Role playing (RP) n = 21	Simulated practice (SP) n = 21
Sex		
Male	7 (33.4%)	3 (14.3%)
Female	14 (66.6%)	18 (85.7%)
University		
University of La Sabana	9 (42.9%)	12 (57.1%)
University of Boyacá	12 (57.1%)	9 (42.9%)
Age	21.4 [2.1]	21.6 [2.3]
Academic average	3.7 [0.3]	3.6 [0.2]
Bilingualism	7.8 [4.2]	8.9 [4.8]
Credits taken	87.2 [13.2]	84.7 [11.6]

$\bar{x}$ [$sd$] = average [standard deviation]

n (%) = absolute frequency (percentage)

Students who had been trained with the SP and RP simulation approach displayed highly significant results in the OCSE-LBP performance. Table 2 shows that in both RP and SP, the final score increased, 0.66 and 0.59, respectively. When comparing the interventions, neither was superior (*p*-value 0.01, 95%CI − 0.21 to 0.23). The study reports 100% participant adherence.

**Table 2:** Comparing the interventions

	Role play				Simulated practice				Difference in SP vs RP^b^	
	Role playing n = 21		Difference in role playing^a^		Simulated practice n = 21		Difference in simulated practice^a^			
	Baseline $$\bar{x}$$$$[s]$$	Post-intervention $$\left[s\right]$$	Diff	95%CI	Baseline $$\bar{x}$$$$[s]$$	Post-intervention $$\left[s\right]$$	Diff	95%CI	Diff	95%CI
Mean OSCE—LBP	2.07 [0.45]	2.73 [0.51]	− 0.66	− 0.97 to − 0.34	2.16 [0.42]	2.76 [0.48]	− 0.59	− 0.89 to − 0.30	0.01	− 0.21 to 0.23

^a^Difference estimated by t-student

^b^Difference estimated by regression model

## Discussion

The study results reveal that pedagogic strategies supported by simulations improve clinical skills in interacting with people with LBP. Both types of simulations generated positive changes in the examination, evaluation, diagnosis, prognosis, and physiotherapeutic intervention. To date, no publications have been found that use simulations to train physiotherapists to interact with people diagnosed with LBP. This could be a powerful tool for enhancing decision-making regarding one of the primary reasons for consulting health services, and high-quality physiotherapy could be a solution for reducing the reoccurrence of this symptom. On the other hand, the nursing profession has registered studies in different clinical conditions that prove clinical reasoning and professional decision-making benefits [42, 43].

Other clinical areas in which physiotherapists work, have already demonstrated the positive effects of simulation to promote other skills. Some antecedents mention that simulations improve teamwork and the understanding of professional roles [44]. Moreover, simulations can replace part of the time in clinical institutions [38], contribute to education in cardiorespiratory physiotherapy [20], improve confidence in students, and enhance the procedural skills in the different performance areas [45], not just in physiotherapy but also in the nursing profession as it has already shown an important effect on anxiety and self-confidence [46].

For performance skills in the musculoskeletal area, Wright et al. [45] reported that after a 100% immersion in clinical simulation for 18 days, students showed significant improvement in their confidence and, in general, in their performance regarding the clinical approach to patients. The authors report that students exposed to rotations in simulated scenarios demonstrated a significantly higher performance in clinical skills than those who did not undergo this immersion.

This study’s simulations were conducted with standardised patients for the SP group and the role-playing scenario in the RP group. Based on the results, both contributed to integrating knowledge developed along the pedagogical process used to achieve the learning objectives related to the therapeutic interaction with a person with LBP. For the SP group, the potential benefit is that direct interaction with others with a specific training can create a similar environment to the reality, which can become an alternative with effects comparable to conventional clinical practices [47]. Despite that both groups showed better clinical reasoning outcomes, Cooper et al. found a more positive perception regarding high fidelity simulations [48]. For the RP group, the literature also reports that it provides students with a better understanding of the patient’s condition and facilitates the integration of knowledge [49].

Although research on clinical simulations as a pedagogic strategy in physiotherapy has increased, it is necessary to continue with reports of this type to enhance student training in therapeutic interactions to improve clinical decision-making [50]. Also, data in the debriefing process should be considered in future studies to better understand the learning experience [51].

The pedagogic process with simulations in both groups (SP and RP) included other educational activities like literature review, retrospective searches about the examination of LBP, laboratory workshops, and classroom sessions. This aspect would be the main limitation of the present study because improvement in the final OSCE-LBP assessment is related to these interventions and not just the simulation activity itself. Additionally, OSCE-LBP has not been psychometrically tested therefore may not have had the ability to detect differences. Other limitations of the study were sample size and the number of sessions on clinical simulation with a simulated patient. Also, the professor that developed the clinical simulation was not blinded.

## Conclusion

From the results herein, including the simulation in the pedagogic process activity resulted in improved decision-making by the physiotherapy students during the process of professional reasoning regarding a person with LBP. As for the design proposed, both types of simulations, RP and SP, were equivalent, both of them can be included like pedagogical practices for training physiotherapy students in LBP treatment. Further research is needed to determine whether this pedagogical process would result in improved physiotherapeutic reasoning skills in real clinical settings. However, this study’s pedagogical intervention material can be implemented in undergraduate physiotherapy education curricula to empower students in clinical reasoning skills for musculoskeletal pain.

## Practice points

- Physiotherapeutic reasoning training in LBP can be combined with simulation scenarios for learning enhancement.
- Role-playing and simulation practice in physiotherapy are teaching strategies that can provide training for musculoskeletal disorders for practitioners of physiotherapy.

## Data Availability

The data collected is available in the public archive of the physiotherapy program of the Universidad de Boyaca.

## Abbreviations

LBP: Low back pain
SP: Clinical simulation with simulated patients
RP: Simulations with role-playing
OSCE-ML: Objective Structured Clinical Examination
