# Correction to: Simulation in physiotherapy students for clinical decisions during interaction with people with low back pain: randomized controlled trial

**DOI:** 10.1186/s12909-021-02859-6

**Published:** 2021-08-09

**Authors:** Carolina Sandoval-Cuellar, Margareth Lorena Alfonso-Mora, Adriana Lucia Castellanos-Garrido, Angélica del Pilar Villarraga-Nieto, Ruth Liliana Goyeneche-Ortegón, Martha Lucia Acosta-Otalora, Rocío del Pilar Castellanos-Vega, Elisa Andrea Cobo-Mejía

**Affiliations:** 1 Universidad de Boyacá, Tunja, Colombia; 2 Universidad de La Sabana, Chía, Colombia; 3 Center of Clinical Simulation, Universidad de La Sabana, Chía, Colombia

**Correction to: BMC Med Educ 21, 375 (2021)**

**https://doi.org/10.1186/s12909-021-02812-7**

Following publication of the original article [1], we have been informed that the author Elisa Andrea Cobo-Mejía has been incorrectly affiliated.

The author affiliation has been updated above and the original article [1] has been corrected.
